# Determinants of consistent condom use among Filipino women: Results from the 2017 Philippine National Demographic and Health Survey

**DOI:** 10.1186/s12889-022-14040-9

**Published:** 2022-08-30

**Authors:** Veincent Christian F. Pepito, Arianna Maever L. Amit, Clinton S. Tang, Ryan Gabriel C. Molen, Luis Miguel B. Co, Neil Andrew Kiamco Aliazas, Sarah J. De Los Reyes, Lourdes Bernadette S. Tanchanco

**Affiliations:** 1 School of Medicine and Public Health, Ateneo de Manila University, Pasig City, Philippines; 2 National Clinical Trials and Translation Center, National Institutes of Health, University of the Philippines Manila, Manila, Philippines; 3 Dr. Fe del Mundo Medical Center, Banawe St., Quezon City, Philippines; 4 The Medical City, Ortigas Ave., Pasig City, Philippines; 5 MedMom Institute for Human Development, Pasig City, Philippines

**Keywords:** Condom use, Consistent condom use, HIV, Unwanted pregnancy, Contraception, Women, Philippines

## Abstract

**Background:**

Consistent condom use in women, defined as the self-reported usage of male condom in every sexual encounter of the respondent with her most recent partner in the last 12 months, had been perennially low in the Philippines. This is despite consistent condom use being a tested and proven public health intervention to prevent unwanted pregnancy and sexually transmitted infections. Considering the high fertility rate, teenage pregnancy rate, and the rapidly increasing incidence of HIV in the country, we identified the determinants of consistent condom use in the Philippines.

**Methods:**

We used data from the individual recode of the 2017 Philippine National Demographic and Health Survey. We used logistic regression for survey data to identify factors associated with consistent condom use.

**Results:**

Out of 25,074 respondents, only 261 (1.13%) have used condoms consistently with their most recent partner. Reach of information and education campaigns on contraceptive use via different media ranged from 62% via television to 7% via short messaging service. After adjusting for confounders, those who were able to ask their partners to use condoms during sexual intercourse have 6.18 times (adjusted odds ratio (aOR): 6.18; 95% Confidence Interval (95% CI): 2.02. 18.94) greater odds of consistent condom use than those who were unable to ask their partners to use condoms during sexual intercourse. Meanwhile, HIV knowledge (aOR: 1.16; 95% CI: 0.98, 1.38) and hearing about contraception in television (aOR: 1.54; 95% CI: 1.00, 2.38) have weaker associations with consistent condom use.

**Conclusions:**

The low percentage of those who use condoms consistently, together with the low reach of information and education campaigns, highlight the need to implement multi-faceted and context-specific interventions to promote sexual agency and/or consistent condom use to address the burden of unwanted pregnancies and HIV in the Philippines.

## Background

High fertility rates, high teenage pregnancy rates, and increasing HIV incidence are three of the most pressing reproductive health issues in the Philippines today. As of 2019, the Philippines has a total fertility rate of 2.98, which is the second highest in Southeast Asia, and is higher than the global average of 2.31 [1]. Similarly, the country has a teenage pregnancy rate of 9% as of 2017, which is one of the highest in Southeast Asia, and has barely changed for the 20 years preceding it [2, 3]. These two demographic issues disadvantage young mothers and their children, and further strains limited government resources and contributes to inter-generational poverty [4]. Lastly, the country is also one of the few countries in the world where the incidence of HIV is rapidly increasing [5]. One of the health interventions that can address these three issues simultaneously is consistent condom use [6, 7].

Despite the established cost-effectiveness of condoms in preventing unwanted pregnancies and sexually transmitted infections (STIs), condom use has always been low in the Philippines. According to the 2017 Philippine National Demographic and Health Survey (NDHS), only 2% of currently married women and 3% of sexually active unmarried women have male partners who have ever used condoms [8]. A systematic review of condom use in the Philippines found 27 studies involving different populations in selected areas throughout the country [9]. The same review has found that the prevalence of consistent condom use ranged from 3% among men who have sex with men in Metro Manila to 74% among female sex workers (FSW) in Iloilo City [9–11]. Furthermore, the same review found that facilitators of condom use include socio-demographic factors such as being single, greater educational attainment, higher monthly wage, longer employment period, type of work establishment, and workplace support. Other facilitators of condom use include attitudes, perceptions and behavioral factors, such as frequent STI tests, higher perceived HIV risk, engaging with FSW, knowledge of HIV, personal decision, positive attitudes on condom use, HIV status, prevention of pregnancies and STIs, peer influence, health provider engagement. Lastly, other factors associated with condom use acting beyond the level of the individual include workplace support, access to health facilities and merchandise stores, and government policies and regulations. On the other hand, barriers to condom use include some personal factors such as perceived discomfort and displeasure, fear, marital status, lack of condoms, low knowledge, religion, and lack of money. Other factors associated with non-use of condoms include attitudinal, perceptional, behavioral risk factors, such as substance abuse, low perceived HIV risk, stigma, use of other contraceptive methods, miscommunication and misinformation, and lack of sex education. Lastly, other factors working beyond the individual level also inhibit condom use such as coercion, preference of partners and peers, discreet placements of condoms in stores and distance to stores, HIV testing requirement, and high price of condoms [9–31]. However, data for most of these studies were collected before the enactment of the Reproductive Health Law and the Executive Order No. 12 in 2012 and 2017, respectively, which both aim to attain and sustain zero unmet need for modern family planning [32, 33]. Moreover, all of the studies have a limited geographic and demographic scope, and only two of them have focused on consistent condom use [14, 16].

In other countries, similar determinants were also found to be associated with consistent condom use [34]. Behavioral skills were found to be associated with consistent condom use among college students in China [35]. In another study involving female sex workers in China, environmental and structural support, perceived benefits and protection, and high safe sex self-efficacy were found to be associated with consistent condom use [36]. In a study involving people living with HIV in Ethiopia, rural residence and partner influence were found to be associated with consistent condom use [37]. In Indonesia, single women, women who lived in urban areas, HIV-positive women, and women involved in sex work have higher odds of using condoms consistently than other women [38].

To address the dearth of nationally representative studies on consistent condom use for the general population which could also serve as basis for further interventions to promote consistent condom use, this analysis aims to examine the determinants of consistent condom use among Filipino women aged 15–49.

## Methods

### Study population and sampling method

The 2017 Philippine NDHS is a nationally representative survey whose primary objective is to provide up-to-date estimates of basic demographic and health indicators. The survey utilized a two-stage stratified design with a Master Sampling Frame by the Philippine Statistics Authority. The strata used were 117 major sampling domains in the country (81 provinces, 33 highly urbanized cities and 3 special areas). The first stage sampling involved the systematic selection of 1,250 primary sampling units throughout the entire Philippines. Such primary sampling units can be *barangays* (Philippine equivalent of village), a portion of a large barangay or two or more adjacent small barangays. Once these primary sampling units were selected, either 20 or 26 housing units were systematically sampled. Non-replacement sampling was utilized to prevent bias. Survey weights were then computed. All women aged 15–49 years old who were either permanent residents of the selected households or visitors who stayed in the households the night before the survey were eligible to be interviewed. Other details of the sampling method utilized by the survey is available in its published manuscript [8].

### Data collection, definition of variables, and data management

Two questionnaires administered using tablet computers by trained field interviewers were used for the 2017 NDHS. The questionnaires were translated to six local languages commonly spoken throughout the Philippines: Tagalog, Cebuano, Ilokano, Bikol, Hiligaynon, and Waray and were then pre-tested. Actual data collection was done on August 14 to October 27, 2017. Data processing began shortly after initiation of data collection, and a clean dataset was prepared by late 2017 [8]. For this analysis, we will use the Individual Recode dataset of the 2017 Philippine NDHS.

The survey collected data on socio-demographic variables, fertility levels and preferences, awareness and use of family planning methods, breastfeeding, maternal and child health, child mortality, awareness and behavior regarding HIV/AIDS, women’s empowerment, domestic violence, and other health-related issues [8]. For this analysis, however, we will only make use of data on socio-demographic, awareness and use of family planning methods, and awareness and behaviors regarding HIV/AIDS. Specifically, the outcome variable for this analysis is consistent condom use, defined as the self-reported usage of male condom in every sexual encounter of the respondent with her most recent partner in the last 12 months. For this variable, we combined the categories for “did not use condom in last sexual intercourse” and “used condom inconsistently” as the no outcome group, while we considered those who used condoms consistently as having the outcome.

From the literature, known determinants of consistent condom use in the Philippines such as educational attainment, employment, HIV knowledge, and alcohol use were included into the resulting regression models [14, 16]. In addition to this, we also assessed the effect of the following variables on consistent condom use: age, wealth index, marital status, HIV testing, tobacco use, intention and usage of traditional and other modern contraceptive methods, total children ever born, husband/partner’s education level, whether it is justifiable for a wife to ask husband to use condom if he has STI, agency (measured as whether the respondent can ask partner to use condom) [39], decision-maker for using contraception, reading about contraception in the internet, heard about family planning on radio, tv, newspaper/magazine, and text message, age of partner, HIV testing, and household size. Age variables were categorized into five-year age-bands to assess the effect of having similar age groups on consistent condom use.

The HIV knowledge questions were aggregated from the answers of respondents on eight yes or no items from the NDHS: (a) reduce risk of getting HIV by having sex with only one partner who has no other partners; (b) reduce risk of getting HIV by always using condom; (c) can get HIV from mosquito bites; (d) can get HIV by sharing food with somebody; (e) a healthy looking person can have HIV; (f) HIV can be transmitted during pregnancy; (g) HIV can be transmitted during delivery; (h) HIV transmitted by breastfeeding. For each item a respondent gets correctly, the respondent gets one point. The points from each item were added to comprise the HIV knowledge score, thus, those who have a higher HIV knowledge score have greater HIV knowledge as compared to those who have low knowledge. We also recoded some categorical variables (e.g., current marital status) to ensure that each category would have sufficient respondents.

### Data analysis

All data management and analyses were carried out in Stata 14.0 IC [40] and we used a level of significance of 0.05 [41]. After data cleaning and recoding, we declared our dataset as survey data using the weighting for the entire survey as sampling weight. All our subsequent analyses are weighted, except for analyses that do not have an option for weighting. Thus, proportions, means odds ratios, and p-values, except for tests of normality and rank-sum tests, were weighted. However, we still showed the number of observations, which are unweighted. Once we declared our dataset as survey data, we ran descriptive statistics for all variables of interest. We identified the proportions and frequencies of the categories for each of our categorical variables. For our continuous variables, we described their range, distribution, and the appropriate measure of central tendency. We also described the number of respondents with missing data for each variable under study.

We then cross-tabulated the different exposure variables with our outcome variable. We noted the p-values of the Pearson’s chi-square test and crude odds ratios. For our continuous exposure variables, we performed either Wald test for normally distributed variables or rank-sum test for skewed variables, to assess their association with the outcome. Once we have done the cross-tabulations, we ran a correlation matrix to assess potential collinearity between variables. If variables had a pairwise correlation coefficient of > 0.70, one of the variables was removed from the analyses. Prior to doing multivariable analyses, we also excluded respondents with those who have missing data in any of the variables of interest by using Stata’s *subpop* function to ensure that estimates and standard errors were computed correctly.

We used logistic regression for survey data to estimate the crude and adjusted estimates of the associations between the different exposure variables and consistent condom use. In choosing the variables to include our final model, we first categorized variables into proximal and distal risk factors. Proximal risk factors are risk factors that are closer to the outcome, while distal risk factors are risk factors that are more “upstream” and usually affect the outcome indirectly. In building our model, we first fit distal factors that are strongly associated with consistent condom use from our cross-tabulations. Once we have the distal factors in our model, we fit the proximal risk factors who were associated with the outcome in our cross-tabulations. For quantitative categorical variables (i.e., age variables), we tested for departure from linearity assumption. Variables which may have unstable estimates due to autocorrelation and/or separation were excluded from the analysis.

## Results

A total of 25,074 women aged 15 to 49 years old, with a mean age of 30.14, from across the Philippines participated in the 2017 Philippine NDHS and were included in the analysis. Around 80% of the population are Roman Catholics, 42% are married, around 86% completed high school or college and 54% reportedly being employed at the time the survey was conducted. Around 51% were reported to be living in rural areas whereas 49% were found in the urban areas. Around three-fourths of respondents did not report alcohol consumption, while 5% reported tobacco consumption. With regards to HIV testing, 95% reported never being tested for the infection despite an average score of 5 in terms of HIV knowledge test. For information regarding family planning and contraceptive knowledge, television was reportedly the most frequent primary source of information (62%), followed by internet (37%), radio (34%) newspaper/magazine (20%) and short messaging service on mobile phone (7%). When asked about intimate relations, the average total lifetime number of sex partners in the observed population was 1.4. Only 261 (1.13%) have reported using condoms consistently with their latest sexual partner in the past 12 months.

Without adjusting for confounding variables, there is strong evidence that educational attainment of respondent, wealth index, current marital status, contraceptive use and intention, belief that wife is justified to ask husband to use condom if he has an STI, agency, hearing information on contraception via internet and/or television, and age group of respondents were strongly associated with consistent condom use (Table 1). In addition to the said variables, HIV knowledge, and total children ever born were also strongly associated with consistent condom use (Table 2).

**Table 1:** Cross-tabulations of categorical exposure variables with consistent condom use

	Inconsistent condom use	Consistent condom use	Missing	p-value	Crude OR (with 95% CI)	p-value of OR
Educational attainment of respondent						
No education	236 (73.87)	0 (0.00)	77 (26.13)	< 0.01	-	
Primary education	3,041 (77.77)	27 (0.84)	785 (21.39)		0.36 (0.20, 0.63)	< 0.01
Secondary education	7,164 (57.89)	102 (0.88)	5,153 (41.23)		0.51 (0.35, 0.73)	< 0.01
Higher	4,694 (53.37)	132 (1.60)	3,662 (45.03)		1	
Missing	0 (0.00)	0 (0.00)	1 (100.00)			
Employment (Respondent currently working)						
No	7,449 (54.72)	112 (0.95)	6,138 (44.34)	0.28	1	
Yes	7,686 (63.86)	149 (1.34)	3,540 (34.80)		1.21 (0.86, 1.72)	0.28
Alcohol consumption						
No	11,185 (57.83)	168 (0.99)	7,543 (41.18)	0.06	1	
Yes	3,950 (62.09)	93 (1.53)	2,135 (36.38)		1.43 (0.98, 2.10)	0.07
Wealth index						
Poorest	4,156 (70.56)	42 (0.62)	1,733 (28.82)	0.01	1	
Poorer	3,494 (64.66)	50 (1.17)	1,947 (34.18)		2.06 (1.17, 3.64)	0.01
Middle	2,924 (61.62)	49 (1.17)	1,883 (37.21)		2.17 (1.28, 3.69)	< 0.01
Richer	2,492 (54.16)	62 (1.21)	2,021 (44.63)		2.54 (1.53, 4.24)	< 0.01
Richest	2,069 (48.22)	58 (1.36)	2,094 (50.41)		3.22 (1.78, 5.84)	< 0.01
Current marital status						
Never in union	480 (5.93)	46 (0.55)	8,126 (93.52)	< 0.01	1	
Married	10,665 (92.64)	170 (1.55)	623 (5.81)		0.18 (0.11, 0.28)	< 0.01
Living with partner	3,779 (94.94)	35 (1.19)	173 (3.86)		0.13 (0.06, 0.30)	< 0.01
Widowed/Divorced/No longer living together or separated	211 (21.60)	10 (1.58)	756 (76.82)		0.79 (0.31, 1.98)	0.61
Ever been tested for HIV						
No	14,573 (58.35)	245 (1.07)	9,457 (40.58)	0.24	1	
Yes	562 (71.27)	16 (2.41)	221 (26.32)		1.84 (0.66, 5.15)	0.25
Tobacco use						
Does not use tobacco	14,306 (58.54)	246 (1.11)	9,344 (40.35)	0.59	1	
Uses tobacco	829 (66.06)	15 (1.50)	334 (32.44)		1.20 (0.63, 2.29)	0.59
Contraceptive use and intention						
Does not intend to use	4,749 (43.11)	10 (0.01)	5,809 (56.84)	< 0.01	1	
Non-user intends to use later	2,456 (38.57)	38 (0.74)	3,580 (60.70)		15.02 (6.75, 33.42)	< 0.01
Using traditional method	1,905 (97.68)	4 (0.31)	49 (2.01)		2.52 (0.46, 13.80)	0.29
Using modern method	6,025 (92.12)	209 (3.61)	240 (4.27)		30.81 (14.61, 64.97)	< 0.01
Religion						
Roman Catholic	10,977 (58.89)	199 (1.19)	6,965 (39.92)	0.17	1	
Protestant	1,413 (58.65)	33 (1.36)	868 (39.99)		1.15 (0.71, 1.85)	0.57
Iglesia ni Cristo	434 (58.24)	6 (0.90)	286 (40.86)		0.77 (0.28, 2.13)	0.61
Aglipay	208 (62.65)	0 (0.00)	127 (37.35)		-	
Islam	1,309 (57.84)	6 (0.34)	1,006 (41.82)		0.29 (0.11, 0.78)	0.02
Other Christian	474 (58.16)	14 (1.14)	295 (40.70)		0.97 (0.50, 1.87)	0.93
Other/None	320 (68.47)	3 (0.55)	131 (30.98)		0.40 (0.11, 1.46)	0.16
Educational attainment of partner						
No education	265 (92.99)	0 (0.00)	14 (7.01)	0.06	-	
Primary education	4,158 (94.22)	35 (1.07)	182 (4.71)		0.52 (0.28, 0.99)	0.05
Secondary education	6,000 (94.30)	85 (1.29)	300 (4.40)		0.63 (0.40, 1.00)	0.05
Higher	4,013 (91.30)	85 (1.98)	300 (6.72)		1	
Missing	699 (7.68)	56 (0.66)	8,882 (91.65)			
Wife justified asking husband to use condom if he has an STI						
No	2,308 (47.35)	23 (0.56)	2,461 (52.09)	0.03	1	
Yes	12,827 (61.40)	238 (1.25)	7,217 (37.34)		1.72 (1.04, 2.85)	0.04
Respondent can ask partner to use a condom						
No	3,893 (95.12)	7 (0.25)	198 (4.63)	< 0.01	1	
Yes	10,551 (92.72)	198 (1.84)	598 (5.44)		7.62 (2.45, 23.70)	< 0.01
Missing	691 (7.62)	56 (0.66)	8,882 (91.71)			
Decision maker for using contraception						
Mainly respondent	1,043 (95.09)	5 (1.57)	41 (3.35)	0.74	1	
Mainly husband/partner	494 (95.25)	14 (3.41)	6 (1.34)		2.17 (0.41, 11.59)	0.37
Joint decision	6,219 (94.71)	168 (2.59)	175 (2.70)		1.66 (0.35, 7.83)	0.52
Other	32 (93.36)	0 (0.00)	2 (6.64)		-	
Missing	7,347 (41.69)	74 (0.48)	9,454 (57.83)			
Read information about contraception on the internet						
No	11,198 (64.94)	157 (1.04)	5,672 (34.02)	< 0.01	1	
Yes	3,937 (48.52)	104 (1.28)	4,006 (50.20)		1.65 (1.13, 2.41)	0.01
Heard family planning on radio last few months						
No	9,181 (55.86)	168 (1.10)	6,846 (43.03)	0.72	1	
Yes	5,954 (64.93)	93 (1.18)	2,832 (33.89)		0.92 (0.60, 1.41)	0.72
Heard family planning on television last few months						
No	5,724 (56.58)	73 (0.84)	4,156 (42.58)	0.04	1	
Yes	9,411 (60.37)	188 (1.31)	5,522 (38.32)		1.46 (1.02, 2.10)	0.04
Heard family planning in newspaper/magazine last few months						
No	12,502 (58.77)	206 (1.05)	8,031 (40.18)	0.21	1	
Yes	2,633 (59.62)	55 (1.46)	1,647 (38.92)		1.37 (0.83, 2.25)	0.22
Heard family planning by test messages on mobile phone						
No	14,243 (59.11)	240 (1.14)	9,101 (39.75)	0.91	1	
Yes	892 (56.46)	21 (1.05)	577 (42.49)		0.97 (0.53, 1.75)	0.91
Domicile (Type of place of residence)						
Urban	5,062 (55.23)	114 (1.26)	3,840 (43.51)	0.06	1	
Rural	10,073 (62.49)	147 (1.01)	5,838 (36.50)		0.71 (0.49, 1.02)	0.07
Age group of respondent						
15–19	525 (10.25)	20 (0.40)	4,575 (89.35)	0.04	1	
20–24	1,926 (45.61)	32 (0.76)	1,956 (53.64)		0.43 (0.22, 0.86)	0.02
25–29	2,707 (71.19)	42 (1.47)	937 (27.34)		0.53 (0.27, 1.05)	0.07
30–34	2,651 (79.59)	58 (1.88)	578 (18.53)		0.61 (0.31, 1.20)	0.15
35–39	2,717 (79.21)	44 (1.81)	530 (18.98)		0.59 (0.25, 1.33)	0.20
40–44	2,353 (80.83)	41 (1.36)	509 (17.80)		0.44 (0.22, 0.87)	0.02
45–49	2,256 (77.00)	24 (0.61)	593 (22.39)		0.20 (0.09, 0.46)	< 0.01
Age group of partner						
15–19	171 (97.52)	6 (2.48)	0 (0.00)	0.23	1	
20–24	1,279 (97.23)	35 (2.77)	0 (0.00)		1.12 (0.37, 3.37)	0.84
25–29	2,161 (98.08)	36 (1.92)	0 (0.00)		0.77 (0.26, 2.29)	0.64
30–34	2,681 (98.35)	42 (1.65)	0 (0.00)		0.66 (0.24, 1.86)	0.43
35–39	2,708 (97.86)	45 (2.14)	0 (0.00)		0.86 (0.27, 2.71)	0.80
40–44	2,436 (97.79)	44 (2.21)	0 (0.00)		0.89 (0.30, 2.63)	0.83
45–49	3,683 (98.79)	51 (1.22)	0 (0.00)		0.48 (0.17, 1.40)	0.18
* Missing*	16 (0.08)	2 (0.01)	9,678 (99.91)

**Table 2:** Continuous variables and their association with consistent condom use

	Range	Mean	Median	Distribution	*p*-value of rank sum test	Crude OR (with 95% CI)	*p*-value of OR
HIV knowledge (*n* = 22,813)	0 – 8	5.18	5	Left-skewed	< 0.01	1.23 (1.06, 1.42)	< 0.01
Total lifetime number of sex partners (*n* = 17,701)	1 – 95	1.40	1	Right-skewed	0.21	0.99 (0.97, 1.01)	0.43
Household size (n-25,074)	1—21	5.39	5	Right-Skewed	0.39	1.02 (0.93, 1.12)	0.65
Total children ever born (*n* = 25,074)	0 – 18	1.77	1	Right-Skewed	< 0.01	0.88 (0.81, 0.97)	< 0.01

Prior to doing multivariable analysis, we assessed for potential autocorrelation between exposure variables. In this assessment, we saw autocorrelation between age group of partner and age group of respondents (r = 0.94). There was also separation when we include contraceptive use and intention in the model. As a result, we no longer consider age group of partner and contraceptive use and intention as exposure variables in the multivariable analysis. Among the exposure variables showing evidence of association with consistent condom use, we identified educational attainment of respondent, wealth index, current marital status, and age group of respondent as distal variables. Alcohol use, employment status, and domicile, while not showing statistically significant relationships with the outcome in the cross-tabulations, were still included in the model as distal variables as they were found to be determinants of the outcome in previous studies [8–30, 37]. The remaining variables showing association with the outcome variable – belief that wife is justified to ask husband to use condom if he has an STI, respondent’s ability to ask partner to use condom, hearing information on contraception via internet and/or television, HIV knowledge, and total children ever born – were classified as proximal variables. We also excluded data from respondents with missing data in any of the remaining variables of interest. As a result, we exclude some 11,791 (47.1%) respondents, mostly those with missing data on consistent condom use or those who have not had any sexual activity yet and are left with 13,283 (52.9%) respondents. Out of these 13,283 respondents, only 200 (1.6%) used condoms consistently with her last partner in the past 12 months.

In building our final model, we assessed whether stratum-specific odds ratio for the age group of the respondent will have to be reported, or a common odds ratio would suffice. However, we found that there was a significant departure from the linearity assumption for the association between age group of respondent and consistent condom use (*p* = 0.05), which means that stratum-specific odds ratios had to be reported. After adjusting for other variables in the model, we find that there is strong evidence of association between agency and consistent condom use (Table 3). Specifically, those who were able to ask their partners to use condoms during sexual intercourse had 6.18 times (adjusted odds ratio: 6.18; 95% Confidence Interval (95% CI): 2.02. 18.94) greater odds of consistent condom use than those who were unable to ask their partners to use condoms during sexual intercourse. Associations between HIV knowledge and hearing about contraception in television with consistent condom use were also borderline significant. A one-point increase in HIV knowledge score translates to 16% greater odds (aOR: 1.17; 95% CI: 0.98, 1.38) of consistent condom use. Meanwhile, hearing about contraception in television translates to 54% greater odds (aOR: 1.54; 95% CI: 0.99, 2.38) of consistent condom use than those who did not hear about contraception via television.

**Table 3:** Association of selected socio-demographic and sexual health variables with consistent condom use (*n* = 13,283)

	Adjusted odds ratio ^*^ (95% CI)	*p*-value
Educational attainment of respondent		
Primary education	1	
Secondary education	0.91 (0.46, 1.82)	0.80
Higher	1.43 (0.70, 2.94)	0.33
Civil Status		
Married	1	
Living with partner	0.87 (0.40, 1.91)	0.73
Wealth index		
Poorest	1	
Poorer	1.54 (0.79, 3.03)	0.21
Middle class	1.49 (0.75, 2.97)	0.26
Richer	1.48 (0.75, 2.94)	0.26
Richest	1.24 (0.58, 2.67)	0.58
Age group of respondent		
15–19	1	
20–24	0.46 (0.06, 3.28)	0.44
25–29	1.10 (0.16, 7.35)	0.92
30–34	1.03 (0.16, 6.78)	0.97
35–39	1.08 (0.15, 7.82)	0.94
40–45	0.83 (0.12, 5.76)	0.85
45–49	0.38 (0.05, 2.81)	0.35
Alcohol consumption		
No	1	
Yes	0.80 (0.45, 1.41)	0.44
Domicile		
Urban	1	
Rural	0.89 (0.60, 1.33)	0.59
Respondent currently working		
No	1	
Yes	1.06 (0.71, 1.57)	0.79
Respondent can ask partner to use condom		
No	1	
Yes	6.18 (2.02, 18.94)	< 0.01
Reading about contraception on the internet		
No	1	
Yes	1.05 (0.68, 1.62)	0.82
HIV knowledge score (common odds ratio)	1.16 (0.98, 1.38)	0.08
Number of children (common odds ratio)	1.07 (0.97, 1.19)	0.17
Respondent believes that it is justified for the wife to ask husband to use condom if he has STI		
No	1	
Yes	1.16 (0.61, 2.21)	0.66
Hearing about contraception on television		
No	1	
Yes	1.54 (1.00, 2.38)	0.05

^*^ adjusted for other variables listed in the table

## Discussion

Despite the proven effectiveness of consistent condom use in preventing unwanted pregnancies and STIs, only 1% of the respondents use condoms with their most recent partner consistently. This could be attributable to the view that condoms are unnecessary because of an assumption of monogamy, as corroborated by the low average number of lifetime sexual partners. However, taken together with the low reach of various campaigns on contraceptive use throughout different forms of media, these findings underscore the need to strengthen sex education and promote optimal reproductive health behaviors, such as consistent condom use, in the general population. After adjusting for other variables, only agency showed a strong evidence of being associated with consistent condom use, which has been demonstrated in a previous study [42]. Other variables, such as HIV knowledge and hearing about contraception in television showed weaker associations with consistent condom use. These variables have been previously identified to be associated with consistent condom use [9–31].

The ability to ask one’s partner to use condoms, a proxy measure for sexual agency, is the only factor strongly associated with consistent condom use. Sexual agency can be defined as the degree to which once can influence the dynamics of a relationship towards a desired outcome. However, because each individual has different experiences and has different environments and context, there are structural inequalities in sexual agency [43]. A qualitative study exploring sexual agency among young women in the Philippines found that many Filipino women have limited to no sexual agency, explaining that their first sexual experience was succumbing to coercion or emotional pressures of being with their partners, or worse, being raped [44]. In another study involving Filipino women, low sexual agency, measured by her inability to ask a partner to use condoms during intercourse, was found to be associated with experiencing intimate partner violence. [39]. Since we find that a woman’s ability to ask a partner to use condoms during intercourse is very strongly associated with consistent condom use, there should be measures to strengthen sexual agency among Filipino women. One scoping review to promote healthy sexuality, which includes sexual agency, is unable to provide a definitive recommendation on what intervention is best implemented to promote healthy sexuality due to different contexts and poor trial design. However, the said review has highlighted the importance of programs and interventions that are multi-faceted and affect not only knowledge and cognition, but also a person’s social and emotional dimensions as well as their well-being [45]. The recent passage of the Philippine Reproductive Health Law, which has specific provisions for adolescent and youth reproductive health guidance and counseling, reproductive health education for adolescents, and prevention and management of reproductive health guidance and counselling, is a step in the right direction [46]. There may be a need to revisit its implementing rules and regulations and further strengthen its implementation considering that inconsistent condom use and teenage pregnancy are still prevalent in the country [8]. A recent paper on sexual agency advocates going beyond focusing solely on sex education interventions in promoting sexual agency. Instead, they advocate for improving living conditions, education, and social support, opining that these have greater effect on improving sexual agency than sex education interventions [47]. The association between sexual agency and consistent condom use also highlights the need for a more holistic campaign to promote modern contraceptive methods, particularly consistent condom use, among older women no longer reached by school-based sex education, by coupling traditional information and education campaigns through television with multi-faceted interventions that aim to improve their living conditions and social support. Peer-led, context-specific community mobilization and advocacy groups are found to have contributed to community collectivization, defined as the belief that collective action can lead to positive change. Community collectivization, in turn, has been shown to promote consistent condom use among FSWs in India [48, 49].

The other variables with weaker associations with consistent condom use – HIV knowledge and hearing about contraception via television – have been shown to be associated with consistent condom use in previous studies as well [9–31]. The association between HIV knowledge and hearing about contraception via television with consistent condom use highlight the need to continue information and education campaigns on HIV via television, as this medium has been shown to be the most effective in encouraging consistent condom use. In information and education campaigns promoting HIV knowledge, the importance of modern forms of contraception, which includes condoms, should be highlighted. However, in doing so, misconceptions on modern contraceptive methods should be addressed [50]. More importantly, a nuanced understanding of how pleasure, power, and inequalities should be considered in devising interventions to promote modern contraceptive methods [51]. The consideration on pleasure is particularly salient in the Philippine context as the perception that condoms are not comfortable to use and causes displeasure has been found to be a barrier to condom use in a previous review. The same review mentions the need for a “collaborative, culturally-sensitive, and population-specific approach to develop and implement acceptable, sustainable and successful condom use interventions” [9]. This study adds that more than just condom use, interventions should focus on promoting consistent condom use as the benefits of preventing STIs and unwanted pregnancies can only come from consistent use of condoms. More importantly, the prevention of teenage pregnancy by consistent condom use also protects women from the stigma and economic hardship brought about by teenage pregnancy. However, it has to be noted that reviews examining the effectiveness of various interventions in promoting condom use, including consistent condom use, have highlighted that there are no definitive interventions that were demonstrated to significantly improve condom use as trials in this field often have very poor quality and behaviors are difficult to change once ingrained [52, 53]. While these reviews noted that there are no definitive interventions that were demonstrated to significantly improve condom use via a direct causal mechanism, community mobilization and activity groups have been shown to improve community collectivization. This, in turn, is associated with consistent condom use among FSWs in India [48, 49].

The lack of significant associations in this study may be due to the low number of people who have the outcome (261 in the descriptive analysis and only 200 left in multivariable analyses). The low numbers of consistent condom users resulted in wide confidence intervals, non-significant result for factors supposedly associated with the outcome (e.g., religion), and residual confounding as we are constrained to select only the strongest confounders to be included in the final model. We were also unable to control for the effect of knowledge of condom use, as this data was not available in the dataset. How the outcome variable is defined also raises possible issues in misclassification. The outcome variable is a combination of two questions – the first one asking if they had used condoms in their most recent intercourse with their most recent partner – and a follow-up question asking if they had used condoms consistently in the past 12 months. This implies that some respondents may be using condoms consistently with their most recent partner, but may not be with their other partners. Alternatively, this could just be an inconsistency in how respondents report their data on contraceptive use and intention, and on condom use, which is quite common in studies using self-report data. Lastly, we have also excluded around half of respondents who have missing data for consistent condom use and/or partner variables, which potentially means selection bias due to missing data and limits the generalizability of our findings to Filipino women who currently have partners in their relationship.

## Conclusion

The low prevalence of consistent condom use in a nationally-representative Philippine survey, together with the low reach of information, education, and communication interventions to promote contraception, highlight the need to strengthen current efforts to promote consistent condom use. After adjusting for confounders, we found that women with greater sexual agency were more likely to use condoms consistently than women with low sexual agency. This implies that interventions to improve sexual agency, such as improving sex education, living conditions, and social support might also work to increase the prevalence of consistent condom use in the population. Such interventions need to be multi-faceted and tailored to our local context and should go beyond traditional information and education campaigns. New policies promoting reproductive health in the Philippines are steps in the right direction but interventions should be implemented in a more impactful manner through multi-faceted programs that affect not only knowledge and cognition, but also a person’s well-being and their social and emotional dimensions. A possible model for interventions to promote consistent condom use are peer-led, context-specific local community mobilization and advocacy groups whose main aim is to promote optimal sexual behaviors, protection from abuse, and uptake of sexual and reproductive health services. Such interventions have been shown to promote community collectivization in India, which, in turn, has been shown to promote consistent condom use among some of their FSWs [48, 49, 54]. Further studies are also needed to study in detail the associations described in the study, and to determine context-specific cost-effective interventions to promote consistent condom use, either through improving sexual agency or through another theory of change, such as community collectivization. With better agency and education, we empower individuals to make responsible choices about their sexual and reproductive health, hopefully addressing the triple burden of teenage pregnancy, high fertility rate, and HIV/STIs in the country.

## Data Availability

The data for the 2017 Philippine National Demographic and Health Survey Individual Recode are available from the Demographic and Health Surveys Program Website (https://www.dhsprogram.com/data/available-datasets.cfm).

## Abbreviations

FSW: Female Sex Workers
HIV: Human Immunodeficiency Virus
MSM: Men who have sex with men
NDHS: National Demographic and Health Survey
STI: Sexually transmitted infection
